# Selection of a Biosafety Level 1 (BSL-1) surrogate to evaluate surface disinfection efficacy in Ebola outbreaks: Comparison of four bacteriophages

**DOI:** 10.1371/journal.pone.0177943

**Published:** 2017-05-22

**Authors:** Karin Gallandat, Daniele Lantagne

**Affiliations:** Department of Civil and Environmental Engineering, Tufts University, Medford, Massachusetts, United States of America; Seconda Universita degli Studi di Napoli, ITALY

## Abstract

The 2014 West African Ebola virus disease outbreak was the largest to date, and conflicting, chlorine-based surface disinfection protocols to interrupt disease transmission were recommended. We identified only one study documenting surface disinfection efficacy against the Ebola virus, showing a >6.6 log reduction after 5-minute exposure to 0.5% sodium hypochlorite (NaOCl) based on small-scale tests (Cook *et al*. (2015)). In preparation for future extensive, large-scale disinfection efficacy experiments, we replicated the Cook *et al*. experiment using four potential BSL-1 surrogates selected based on similarities to the Ebola virus: bacteriophages MS2, M13, Phi6, and PR772. Each bacteriophage was exposed to 0.1% and 0.5% NaOCl for 1, 5, and 10 minutes on stainless steel. MS2 and M13 were only reduced by 3.4 log and 3.5 log after a 10-minute exposure to 0.5% NaOCl, and would be overly conservative surrogates. Conversely, PR772 was too easily inactivated for surrogate use, as it was reduced by >4.8 log after only 1-minute exposure to 0.5% NaOCl. Phi6 was slightly more resistant than the Ebola virus, with 4.1 log reduction after a 5-minute exposure and not detected after a 10-minute exposure to 0.5% NaOCl. We therefore recommend Phi6 as a surrogate for evaluating the efficacy of chlorine-based surface disinfectants against the Ebola virus.

## Introduction

The Ebola virus is a filamentous, enveloped, single-stranded RNA virus belonging to the *Filoviridae* family [1]. It was isolated in 1977 following an outbreak in Zaire (now the Democratic Republic of Congo) [2] and the 2014 West African Ebola Virus Disease (EVD) outbreak was the largest to date, with over 28,000 cases and 11,000 deaths [3].

The primary transmission pathways for EVD are in direct contact with infected individuals and their bodily fluids, particularly in caring for a patient in the late stage of the disease and in unsafe burials; contact with bodily fluid-contaminated surfaces is a secondary transmission pathway [4–7]. The Ebola variant isolated during the 2014 outbreak (Makona-C05) was found to persist longer on surfaces than the Yambuku-Mayinga variant from 1976, representing increased risk of disease transmission via contaminated surfaces [8].

Agencies involved in EVD response have developed guidelines for disinfection of surfaces [9–12] and hands [12,13]] to prevent disease transmission in Ebola Treatment Units (ETU) and communities impacted by EVD. While these guidelines are consistent in recommending disinfection of surfaces (“non-living things”) with 0.5% chlorine solutions and disinfection of hands (“living things”) with 0.05% chlorine solutions, they differ in terms of exposure time and recommended practices (e.g. whether or not to pre-clean or cover uncontrolled spills from patients). During the 2014 outbreak, it became clear that these guidelines were not evidence-based. One reason for the lack of evidence is that testing using the Ebola virus can only be performed in BSL-4 facilities and is thus limited [14].

Surrogates are often selected to model highly infectious pathogens [15], and identification of surrogates for the Ebola virus was listed as a priority research item in 2015 [14]. Bacteriophages are commonly used as surrogates for human viruses, as they are similar in terms of size, shape, morphology, surface properties, mode of replication, and environmental persistence, yet are non-infectious [16]. Additionally it is possible to complete large sample size investigations with bacteriophages, as testing is rapid and inexpensive.

The ideal bacteriophage surrogate would behave similarly to the Ebola virus when exposed to surface disinfection with chlorine. Enveloped viruses, like Ebola, had previously been thought to be more susceptible to environmental conditions, and as such disinfectants, than non-enveloped viruses [17]. Current guidelines still recommend using disinfectants whose efficacy was demonstrated against non-enveloped viruses for surface disinfection of enveloped viruses, based on this assumption [18]. However, more recent research has called this assumption into question [19], and the fundamental mechanisms by which chlorine inactivates viruses remain incompletely understood [20,21]. Given this incomplete understanding of how chlorine inactivates viruses, it is not possible to select an appropriate surrogate for the Ebola virus in the absence of experimental data.

While persistence of the Ebola virus on surfaces has been extensively studied [8,22,23], few published data are available regarding the resistance of the virus to disinfecting agents on surfaces [24–27]. The study most relevant to field disinfection practices on surfaces was conducted by Cook *et al*. [24], who evaluated the survival of the Ebola virus when exposed to chlorine on stainless steel surfaces. After a 10-minute exposure to 0.1% sodium hypochlorite (NaOCl), a 2.8 log reduction was observed and the Ebola virus was not detected after a 5-minute exposure to 0.5% NaOCl. These results provide a basis for comparison of potential surrogates with the actual Ebola virus.

In this study, we replicated the Cook *et al*. experiment [24] with four potential bacteriophage surrogates to determine which one would be the most appropriate surrogate for evaluating the efficacy of surface disinfection with chlorine in inactivating the Ebola virus. Our goal was to select a bacteriophage that was slightly more resistant to surface disinfection with chlorine than the Ebola virus as a surrogate for our future studies.

## Methods

**Fig 1:**
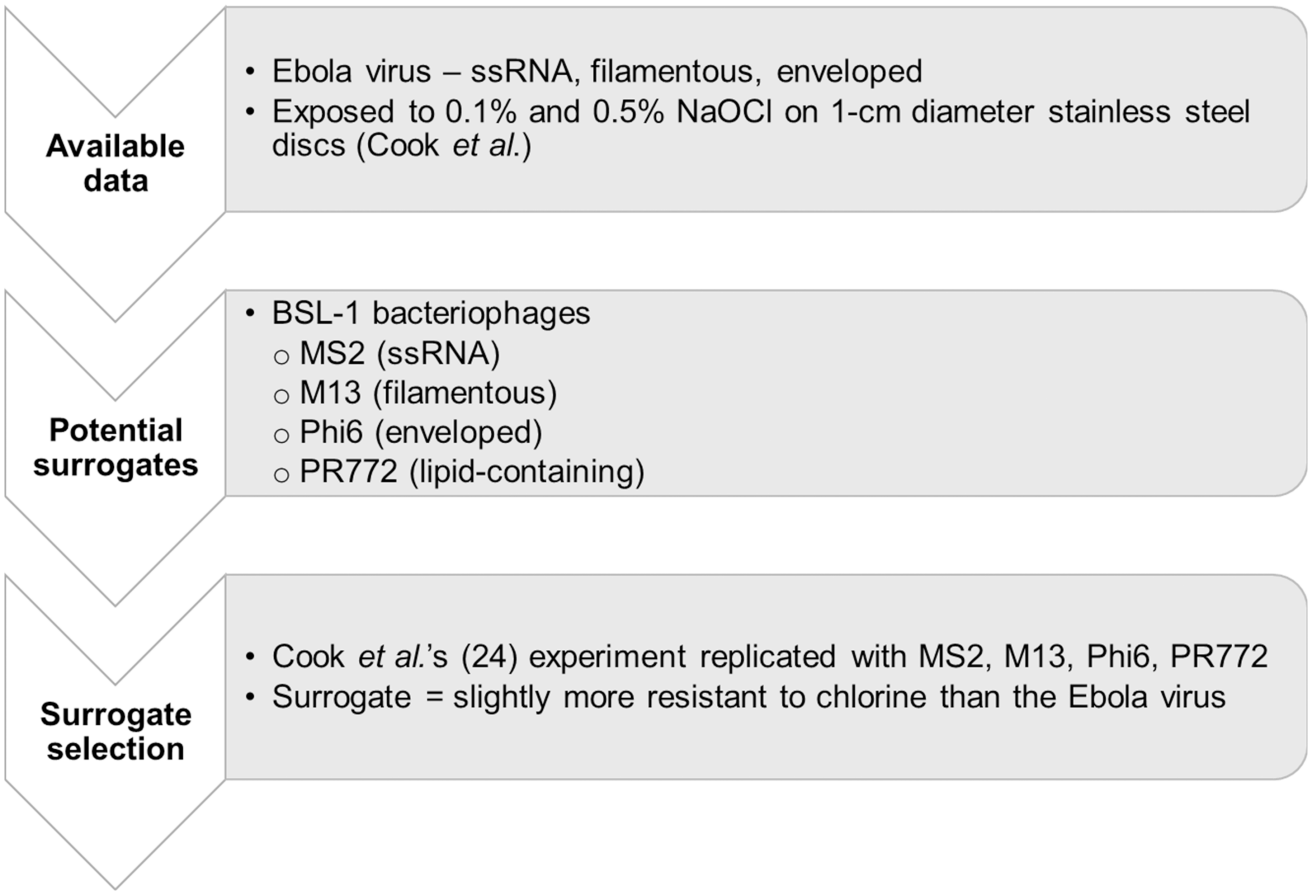
Approach for the selection of a bacteriophage surrogate for the Ebola virus to evaluate surface disinfection efficacy.

To complete this work, we reviewed potential surrogates and selected them; propagated the selected surrogates in the Environmental Sustainability Laboratory at Tufts University (Medford, MA, USA); replicated the experiment described by Cook *et al*. [24] for each of the selected surrogates; and, completed data analysis. The overall process is summarized in Fig 1.

### Selection of potential surrogates

We used the Sinclair *et al*. [15] framework to select potential surrogates for the Ebola virus. In particular, after determining the scope of our experiments—i.e. chlorine disinfection of surfaces–, we considered both practical and biological attributes to identify potential surrogates. Practical considerations initially led us to focus on bacteriophages, as described in introduction. We then used three main criteria inspired from Sinclair *et al*. [15] to identify potential surrogates for the Ebola virus: safety, functional morphology and expected resistance to chlorine. As we planned to conduct further research on surface disinfection and handwashing efficacy with human subjects using the selected surrogate, we only considered bacteriophages with BSL-1 hosts. We selected four bacteriophages, of which three share characteristics with the Ebola virus, namely MS2, M13, and Phi6 (Table 1). The fourth bacteriophage is the lipid-containing PR772. Because the role of the envelope in determining the resistance of viruses to disinfection might be important, we would ideally have included a second enveloped bacteriophage to test that hypothesis. However, Phi6 belongs to the *Cystoviridae*, which is the only known family of enveloped bacteriophages [28] and members of the same family would be too similar to assess the possible role of the envelope based on survival data. We therefore used PR772 instead, which has an internal, rather than an external, lipid structure. Each of the selected bacteriophages is further described below.

**Table 1:** Characteristics of the Ebola virus and of the selected test organisms.

	Filamentous	Genome type	Genome size	Envelope	Capsid
**Ebola virus**	Yes	(-)ssRNA	18 kb	Yes	Helical
**MS2**	No	(+)ssRNA	3.5 kb	No	Icosahedral
**M13**	Yes	ssDNA	6.4 kb	No	Helical
**Phi6**	No	dsRNA	13 kb	Yes	Icosahedral
**PR772**	No	dsDNA	15 kb	No	Icosahedral

MS2 is a single-stranded RNA (ssRNA) bacteriophage, with the same genome type as the Ebola virus. MS2 is commonly used in environmental studies as a model for enteric viruses and is known to be resistant to several disinfection processes [20,29]. As such, MS2 was expected to be a conservative surrogate for the Ebola virus.

M13 is filamentous with a helical capsid, morphologically similar to the Ebola virus. M13 has an average length of 900 nm with a diameter of 6–10 nm [30], and the Ebola virus is about 805 nm long with a diameter of 80 nm [31]. We found no information on the susceptibility of M13 to surface disinfection with chlorine.

Phi6 is enveloped, like the Ebola virus. It has been used to study the fate and partitioning of enveloped viruses, and of the Ebola virus in particular, in sewage [32–34]. Phi6 was also proposed as a slightly conservative surrogate for the H5N1 avian influenza virus when testing liquid chlorine disinfection [35]. Phi6 was expected to be more susceptible to chlorine than MS2 and M13.

PR772 is a lipid-containing bacteriophage that is genetically 97.2% similar to PRD-1 but does not require a BSL-2 host. PRD1-like phages are frequently used in environmental studies and thought to be appropriate surrogates for adenoviruses [16]. PR772 has an internal lipid structure, but no external envelope, so we expected it to be more resistant to chlorine than Phi6 based on morphology. Previous studies on liquid chlorine disinfection [35,36] suggest however that PR772 would be less resistant to chlorine than Phi6.

### Phages propagation

The test organisms were: MS2 (ATCC^®^ 15597-B1) and M13 (ATCC^®^ 15669-B1), both using *Escherichia coli* (ATCC^®^ 15597) as a host; PR772 (HER #221) propagated in *Escherichia coli* (HER #1221); and, Phi6 (HER #102) propagated in *Pseudomonas syringae* (HER #1102). All bacteriophages were propagated by the double agar overlay method [37]. The media were ATCC^®^ #271 for MS2/*E*. *coli*, ATCC^®^ #274 for M13/*E*. *coli*, Nutrient Broth Yeast Extract for Phi6/*P*. *syringae*, and Tryptic Soy for PR772/*E*. *coli*. All *E*. *coli* cultures and MS2, M13 and PR772 were incubated at 35°C, while Phi6 and its host were incubated at 26°C. For all bacteriophages, 100 μL of host culture and 100 μL of phage suspension were added to 6 mL of melted soft agar (0.3%) that were then poured onto prepared agar plates (1.5%) and incubated overnight. On the following day, 5 mL of dilution buffer were applied on top of the soft agar and left at room temperature for 4 hours to allow diffusion of the phages, as described in Rossi [38]. The phage-containing buffer was then recovered, filtered at 0.22μm and stored at 4°C until use. Bacteriophage stocks were not purified because the recovered suspension was free of cellular debris [38] and because soil load was added in a further step to increase chlorine demand. The chlorine demand of the phage suspension alone represented less than 0.01% of the minimum amount of chlorine available for disinfection in our experiment.

### Replication of Cook *et al*.’s experiment

To replicate the Cook *et al*. [24] experiment, we first prepared chlorine solutions, soil load, and surface carriers; and then completed the experimental procedure.

Solutions of 0.1% (1,000 mg/L) and 0.5% (5,000 mg/L) chlorine were prepared by diluting 5.25% laboratory-grade pH-stabilized bleach (Valtech, Zellenople, PA, USA) in hard water (0.04% CaCO_3_). The chlorine concentration was confirmed to be within 10% of the target 0.1% or 0.5% concentration by iodometric titration (Hach method #8209, Hach Company, Loveland, CO, USA). A soil load containing 7.80 mg/mL bovine serum albumin (Sigma-Aldrich, St. Louis, MO, USA), 2.52 mg/mL bovine mucin (Alfa Aesar, Ward Hill, MA, USA), and 10.92 mg/mL tryptone (FisherScientific, Fairlawn, NJ, USA) was prepared according to the ASTM International Quantitative Carrier Testing standard [39]. Surface carriers were 1-cm in diameter discs of type 430 brushed stainless steel (McMaster Carr, Atlanta, GA, USA) that were sterilized by autoclaving and placed in inverted 18-mm test tube caps.

Ten microliters of the phage/soil load mixture were applied onto each disc with a positive-displacement pipette and left to dry in a biosafety cabinet at room temperature and approximately 10% relative humidity. After one hour, 50 μL of a 0.1% or 0.5% NaOCl solution were applied for 1, 5, or 10 minutes. At the end of the exposure time, 950 μL of 0.25% sodium thiosulfate solution were added to neutralize the chlorine. For the positive controls, which correspond to time point zero, 1,000 μL of 0.25% sodium thiosulfate solution were added to the carriers. The samples were vortexed, recovered by pipetting, and titrated by spotting 50 μL drops on top of the soft agar layer containing 100 μL of the bacterial host culture and incubating overnight at the appropriate temperature. All tests were performed in triplicate. Tests with 0.1% and 0.5% NaOCl were performed at the same time for each bacteriophage, with one series of triplicate positive controls (without chlorine) for both concentrations.

### Data analysis

Cook *et al*. [24] kindly shared their numeric data to allow for a direct comparison of the potential surrogates with the Ebola virus. Our data was entered and analyzed in Microsoft Excel^®^ 2016 (Redmond, WA), including calculation of average log reductions, first-order inactivation constants, and standard errors. As the theoretical detection limit was 20 PFU/mL, samples with zero detected bacteriophage were assigned a value of 10 PFU/mL for all calculations. We did not perform any statistical test to support that selection because we felt that this would be an over-analysis of a small data set (triplicates of one representative experiment only for each bacteriophage) [40].

## Results

The surface carriers were inoculated with 10 μL of suspensions containing an average of 6.8∙10^7^ PFU/mL for MS2, 3.6∙10^8^ PFU/mL for M13, 1.2∙10^8^ PFU/Ml for Phi6, and 5.0∙10^9^ PFU/mL for PR772. The recovery rates, as estimated based on the positive controls after 1-hour drying, were on the order 0.8% for MS2, 2.1% for M13, 0.4% for Phi6, and 0.01% for PR772. The starting concentrations (time zero for disinfection, determined based on positive controls) were thus 5.7∙10^5^ PFU/mL for MS2, 7.7∙10^6^ PFU/mL for M13, 5.1∙10^5^ PFU/mL for Phi6, and 6.4∙10^5^ PFU/mL for PR772.

**Fig 2:**
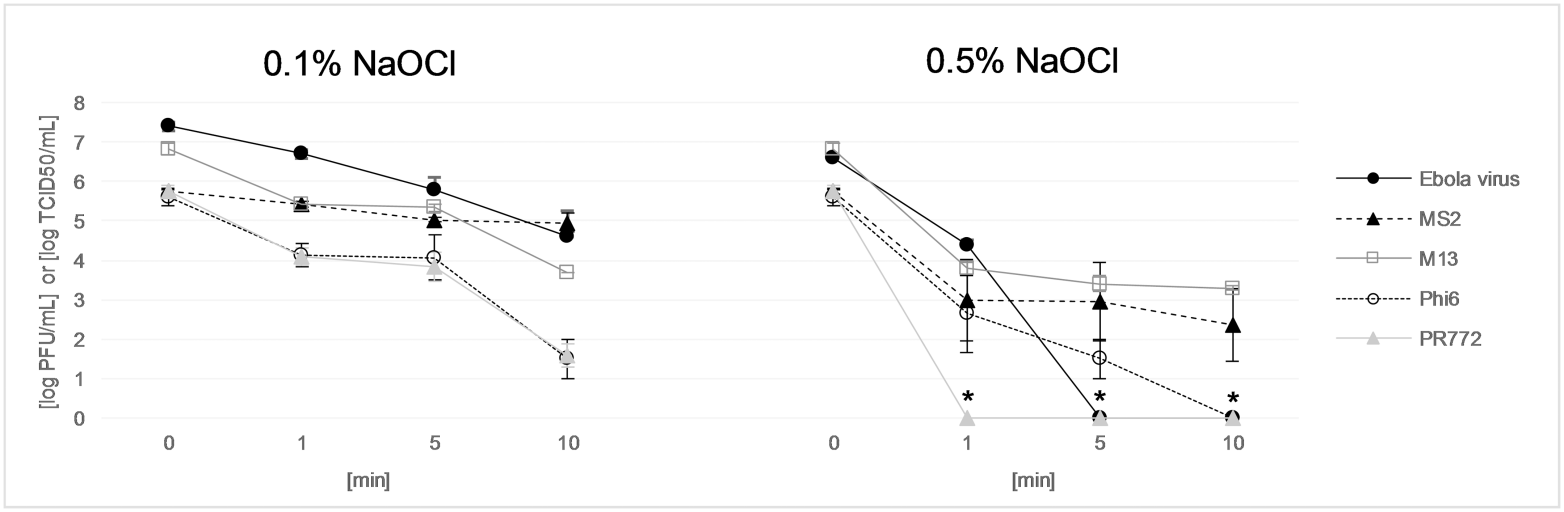
Behavior of bacteriophages MS2, M13, Phi6, and PR772 and of the Ebola virus (results from Cook *et al*. [24]) when exposed to 0.1% and 0.5% NaOCl for 1, 5, and 10 minutes. The units are [log_10_ PFU/mL] for the bacteriophages and [log_10_ TCID50 units/mL] for the Ebola virus. The error bars represent the standard error of the mean of triplicate experiments (3 biological replicates of 3 technical repetitions for the Ebola results). The asterisks indicate that concentrations were under the detection limit (for PR772 at 1, 5, and 10 minutes, and for the Ebola virus at 5 and 10 minutes).

At 0.1% NaOCl (Fig 2), the first-order inactivation constants (k) reflect the fact that after an initial steep decline during the first minute of exposure to chlorine, all four bacteriophages undergo relatively slow inactivation from 1 to 5 minutes, with k values between 0.03 and 0.23 min^-1^ (Table 2). Past 5 minutes of exposure, while k values are similar for M13 (1.17 min^-1^), Phi6 (1.18 min^-1^) and PR772 (1.03 min^-1^), the inactivation constant for MS2 is only 0.04 min^-1^. In comparison, the corresponding k value for the Ebola virus (calculated based on Cook *et al*.’s results at 1 and 5 minutes) is 0.61 min^-1^.

**Table 2:** Observed average first-order inactivation constants (k, min^-1^) and log inactivation (LI) at 0.1% and 0.5% NaOCl.

NaOCl	Time [min]	Ebola virus		MS2		M13		Phi6		PR772	
		k	LI	k (SE)	LI (SE)	k (SE)	LI (SE)	k (SE)	LI (SE)	k (SE)	LI (SE)
0.1%	0–1	0.41	0.7	0.73 (0.10)	0.32 (0.06)	3.24 (0.49)	1.41 (0.35)	3.41 (0.41)	1.48 (0.29)	3.85 (0.28)	1.67 (0.21)
	1–5	0.55	1.6	0.23 (0.02)	0.71 (0.06)	0.05 (0.11)	1.50 (0.10)	0.03 (0.19)	1.54 (0.57)	0.16 (0.12)	1.94 (0.37)
	5–10	0.61	2.8	0.04 (0.08)	0.79 (0.23)	1.17 (0.32)	3.13 (0.34)	1.18 (0.17)	4.11 (0.50)	1.03 (0.11)	4.18 (0.29)
0.5%	0–1	5.16	2.2	6.34 (1.19)	2.75 (1.04)	7.10 (0.28)	3.05 (0.15)	6.83 (1.14)	2.97 (0.97)	10.98 (0.14)	>4.77 (0.00)
	1–5	2.65	>6.6	-0.03^a^ (0.38)	2.80 (0.98)	0.17 (0.05)	3.42 (0.08)	1.11 (0.19)	4.11 (0.49)	N/A	>4.77 (0.00)
	5–10	N/A	>6.6	0.27 (0.31)	3.39 (0.91)	0.08 (0.04)	3.53 (0.14)	1.41 (0.13)	>4.61 (0.00)	N/A	>4.77 (0.00)

Data for the Ebola virus are based on Cook *et al*. [24]. SE stands for standard error of the mean. N/A corresponds to cases where the k constant could not be calculated because of consecutive samples being under the detection limit.

^*a*^ The negative value is due to the detection of a slightly higher concentration at 5 minutes compared to 1 minute in some samples. This was possible because all samples were sacrificial, i.e. different discs were inoculated to evaluate disinfection efficacy at 1, 5 and 10 minutes.

When exposed to 0.5% NaOCl, PR772 was never detected after disinfection (Fig 2), which corresponds to an inactivation constant of 10.98 min^-1^ (Table 2). From zero to 1 minute, the inactivation constants for MS2 (6.34 min^-1^), M13 (7.10 min^-1^) and Phi6 (6.83 min^-1^) are all slightly higher than for the Ebola virus (5.16 min^-1^); from 1 to 5 minutes, however, the average k values for MS2 (-0.03 min^-1^) and M13 (0.17 min^-1^) indicate that they are much more resistant to chlorine than the Ebola virus (2.65 min^-1^), while the k value for Phi6 (1.11 min^-1^) is the closest to, although smaller, than that of the Ebola virus.

## Discussion

We evaluated four potential bacteriophages for their appropriateness as a BSL-1 surrogate for assessing the efficacy of chlorine in inactivating the Ebola virus on stainless steel surfaces. Based on our results at the 0.5% NaOCl concentration recommended for surface disinfection in EVD outbreaks, Phi6 was selected as the most appropriate surrogate as it had slightly conservative results when compared to the Ebola virus. MS2 (as expected) and M13 were too conservative and PR772 was too rapidly inactivated for use as a surrogate. At 0.1% NaOCl, MS2 is the only bacteriophage that is more resistant than the Ebola virus; however, given their similar behavior, all tested bacteriophages could be used as surrogates.

Our recovery rates were low compared to the 24–76% reported by Tuladhar *et al*.[41] using methods similar to ours. The difference is likely due to the higher relative humidity in their case (40–45%). Herzog *et al*.[42], who used wipes to sample bacteriophages P22 from 100-cm^2^ fomites, documented recovery rates <3% after 20 minutes drying and found that recovery rates decreased with decreasing relative humidity.

Few studies have been published that provide data allowing for a comparison with our results. Morin *et al*. [29] reported >4 log reduction for MS2 when exposed to 0.1% NaOCl for 15 minutes on stainless steel carriers. In addition to the longer contact time, the fact that they used larger surface carriers (20x10x1mm) and spread the 10-μL “spill” to cover 70% of the surface before drying for 1 hour, enhancing the impact of drying, might explain their increased inactivation compared with our results.

In a study of Phi6 inactivation by chlorine in the liquid phase, Adcock *et al*. [35] observed a 3 log reduction following exposure to 0.56 mg Cl_2_∙min/L at 5°C and pH 7.0. In contrast, Gall *et al*. [36] recently reported >6 log reduction for PR772 when exposed to 0.5 mg Cl_2_∙min/L in the liquid phase, at 1°C and pH 8.7, and were not able to determine kinetics at lower pH due to rapid inactivation of PR772. Although these studies evaluated the resistance to disinfection in the liquid phase, they confirm that PR772, with its internal lipid structure, is more sensitive to free chlorine than the enveloped Phi6. Further research is needed to understand what might be the underlying mechanisms.

While all bacteriophages behave similarly when exposed to 0.1% NaOCl, they exhibit strikingly different inactivation rates when the chlorine concentration increases to 0.5%: MS2 and M13 undergo a very slow inactivation past the first minute of exposure, while Phi6 and PR772 are inactivated more quickly than at 0.1% NaOCl (as expected). This might reflect the fact that the chlorine demand of the soil load and virus mixture is proportionally more important at 0.1% compared to 0.5% NaOCl, and drying might have a stronger influence on the observed survival at the lower concentration.

Our study has limitations. We intended to replicate the Cook *et al*. [24] experiment but laboratory conditions may have been unintentionally different. In particular, the relative humidity in our laboratory was lower than that reported by Cook *et al*. Conflicting information is found in the literature regarding the effect of relative humidity on the survival of viruses on surfaces [43,44], and it remains unclear whether and how enhanced drying would affect the resistance of test organisms to chlorine. The time zero bacteriophage concentrations were slightly lower than the Ebola concentrations, partly due to low recovery rates. While comparison of each bacteriophage to the Ebola virus was primarily based on the inactivation rate, having different starting points makes the comparison of log inactivation difficult and we cannot rule out that some of the observed differences could be due to variability in recovery. Please note our data do not support the use of Phi6 as an Ebola virus surrogate in conditions different than those described above.

Our conclusion that Phi6 is an appropriate and slightly conservative surrogate for the Ebola virus when testing chlorine disinfection on stainless steel might be extrapolated to other surfaces if surface type does not impact the virus-disinfectant interaction; further research is needed to determine whether this assumption is valid. Additionally, future research investigating the viral inactivation mechanisms underlying disinfection, and how surface chemistry and drying might interact with disinfection processes, is recommended. A more fundamental understanding of viral inactivation mechanisms will provide stronger support for the selection of appropriate surrogates for highly infectious pathogens.

Lastly, we recommend continued development of partnerships between BSL-4 and non-BSL-4 laboratories to: 1) assist in evidence-based surrogate selections; and, 2) conduct a broad range of experiments with surrogates and then downselect critical conditions to test with actual pathogens in BSL-4 conditions.
